# Platforms for Single-Cell Collection and Analysis

**DOI:** 10.3390/ijms19030807

**Published:** 2018-03-11

**Authors:** Lukas Valihrach, Peter Androvic, Mikael Kubista

**Affiliations:** 1 Laboratory of Gene Expression, Institute of Biotechnology CAS, Biocev, Vestec 252 50, Czech Republic; lukas.valihrach@ibt.cas.cz (L.V.); peter.androvic@ibt.cas.cz (P.A.); 2 Laboratory of Growth Regulators, Faculty of Science, Palacky University, Olomouc 783 71, Czech Republic; 3 TATAA Biocenter AB, Gothenburg 411 03, Sweden

**Keywords:** single cell, collection, isolation, analysis

## Abstract

Single-cell analysis has become an established method to study cell heterogeneity and for rare cell characterization. Despite the high cost and technical constraints, applications are increasing every year in all fields of biology. Following the trend, there is a tremendous development of tools for single-cell analysis, especially in the RNA sequencing field. Every improvement increases sensitivity and throughput. Collecting a large amount of data also stimulates the development of new approaches for bioinformatic analysis and interpretation. However, the essential requirement for any analysis is the collection of single cells of high quality. The single-cell isolation must be fast, effective, and gentle to maintain the native expression profiles. Classical methods for single-cell isolation are micromanipulation, microdissection, and fluorescence-activated cell sorting (FACS). In the last decade several new and highly efficient approaches have been developed, which not just supplement but may fully replace the traditional ones. These new techniques are based on microfluidic chips, droplets, micro-well plates, and automatic collection of cells using capillaries, magnets, an electric field, or a punching probe. In this review we summarize the current methods and developments in this field. We discuss the advantages of the different commercially available platforms and their applicability, and also provide remarks on future developments.

## 1. Introduction

Single-cell analysis has become an attractive and challenging field of modern molecular biology and medicine, the main goal of which is to study biological questions with single-cell resolution [1,2,3]. Such an approach reflects cell heterogeneity and reveals the complex response of an organism to various physiological and pathophysiological stimuli [4,5,6,7,8,9]. Another important application is the analysis of rare cells, such as circulating tumor cells (CTC), residual cells relevant to disease or therapy, and stem cells [10,11,12,13,14,15,16]. The ability to characterize rare cells is important in diagnosis and prognosis of disease, but also for the understanding of key regulatory mechanisms distinguishing the development of normal cells from those undergoing pathological processes [3,17,18]. For these reasons, single-cell analysis has become one of the most interesting topics in contemporary biology and a rapidly growing field within the life sciences [2,19,20,21].

To properly describe and understand the complexity of the biological systems, genetic regulation must be studied on all levels, including DNA, transcription of mRNAs and different regulatory RNAs such as microRNAs and other non-coding RNAs, proteins, cell metabolites, hormones, etc., [19,21]. For each type of target analyte there are also several approaches applicable on the single cell level [4,22,23,24]. Multianalyte analysis in individual cells has already been described [25,26,27]. Since the concentration of analytes is usually very low in individual cells, the basic requirements of any method are high sensitivity and specificity, preferably with a multiplex option. The most commonly used techniques are quantitative PCR (qPCR), RT-qPCR, and RNA/DNA sequencing (RNA/DNA-Seq) for nucleic acids, and immunohistochemistry (IHC) and quantitative mass spectroscopy (MS) for proteins [28,29,30,31]. In particular, RNA-Seq in single cells is a hot topic with new approaches that increase throughput and reduce costs emerging frequently [18,19,21,31].

Despite progress in the quantification of target molecules, the collection of single cells of high quality with minimally perturbed native expression profiles remains challenging [19,21,23,32]. Several methods, approaches, and instruments for single-cell collection are available, each with its advantages and limitations (time, throughput, price, spatial resolution, etc. [17,18,22,33]), and more are under development (e.g., digital droplet PCR-based (ddPCR) platform from Stilla Technologies (personal communication)). To sum up the current state of the art, we review the most recent single-cell isolation platforms. We compile the basic principles, features, and potential applications of each to provide a comprehensive overview. Future perspectives on single-cell isolation and analysis are also discussed. Traditional micromanipulation, fluorescence-activated cell sorting, and laser capture microdissection methods have already been reviewed in detail elsewhere [34,35,36,37,38,39,40,41,42,43] and are only briefly discussed.

## 2. Identification of Cells of Interest

A fundamental requisite to collect cells of interest is their identification within the heterogeneous population. The selection of cells of a certain type is often based on fluorescent labeling, either directly by a fluorescent antibody or by the expression of a protein genetically engineered to be fluorescent (green, yellow, or red) and specifically expressed in the targeted cell type [44,45,46]. The limitations of the first approach are available antibodies (particularly for less common organisms), cross-reactivity to other targets, and background unspecific labeling [47,48,49]. The second approach avoids many of these issues, but the identification of a suitable marker gene may be very complicated, especially under pathological conditions, when expression of cells is boosted and the presumptive marker can also appear in other cell types [50,51,52]. The selection of markers and the application of antibodies against them are also limited when living cells dissociated from tissues are analyzed. Only surface proteins can then be targeted, since antibodies generally do not penetrate cell membranes. Undesired digestion of surface proteins that could serve as markers by enzymes used in the dissociation procedure is often also an issue [53,54]. Extending the recovery time of cells after dissociation to allow for re-synthesis of surface markers entails a risk of perturbing the expression profiles [55,56]. Another approach is to select cells based on morphological criteria such as size, shape, or position within the tissue, or other properties of the targeted cells, such as electrophysiological behavior [22,57,58].

## 3. Traditional Approaches for Single-Cell Collection

Traditional methods for single-cell isolation are micromanipulation, fluorescent-activated cell sorting (FACS), and laser capture microdissection (LCM). These are well-established and standardized techniques with a diverse spectrum of applications [22,23,33,34,35,38]. The first two allow for the collection of live cells from dissociated tissues or in vitro cultures. The last technique usually requires fixation of the tissue or cells, which causes cell death, although some LCM protocols to analyze live cells have been described [59,60]. The throughput of the methods is also quite different. While FACS is extremely efficient in cell sorting and thousands of cells can be sorted in a very short time, micromanipulation and LCM are very slow and laborious techniques, allowing only very limited throughput [23]. The time needed to collect a cell is the most important factor that influences changes in gene expression. Expression profiles can also be altered by the dissociation procedure used [56]. The degree of change depends on many factors including protocol, type and concentrations of reagents used, incubation time, temperature, etc. Least variable in this sense is the LCM approach, where RNA is stabilized using fixatives. However, these promote RNA degradation, influencing the quality of the data and possibly also introducing bias [61,62,63]. Although formalin-free fixatives are available, the RNA quality may still be affected [64,65]. An important advantage of LCM is spatial information about the cells in the tissue. Such information can be biologically most relevant and is lost when tissues are dissociated. Another advantage is that any remaining tissue can be stored for later. The disadvantage is possible contamination by fragments of neighboring cells, if the studied cell is not cut out precisely [33,60]. Contamination can also be an issue when cells are collected by micromanipulation, when a small volume of medium is co-transferred with the selected cells [23]. Analyzing negative control samples is therefore highly recommended in single-cell applications. To avoid contamination, a narrower tip of a patch clamp pipette can be used to harvest only cytosol medium. This approach can also be applied for simultaneous measurement of other features of the cell of interest, e.g., the electrophysiological properties of cell membranes [66,67,68]. This method is particularly useful when studying excitable cells such as neurons, glial cells, cardiomyocytes, etc. Multiple parameters can also be analyzed using FACS (up to 50 markers simultaneously by using element labels with inductive coupled plasma (ICP) mass spectrometric detection [34,69,70]). Another important factor when collecting single cells is cell morphology. While in FACS morphological information is limited to cell size (forward scatter) and complexity (side scatter), micromanipulation and LCM allow for visual inspection of the cells. High-resolution pictures of each cell can be captured and used to control for quality [19]. The three approaches also differ in the amount of cells needed for analysis. While micromanipulation and LCM may be used on a limited number of cells, FACS requires large amounts. Being able to start with a very large number of cells may be advantageous when rare cells are sought [3,29]. The three traditional approaches for single-cell collection are summarized in Table 1.

Cells of interest are usually harvested directly into a lysis buffer that is compatible with downstream molecular analyses [71,72], including incorporation of barcoded oligonucleotides for single-cell RNA-Seq or lanthanide-based barcoding for MS. The current trend is to integrate collection and analysis of the single cells in one instrument [32,73,74,75]. Most popular is single-cell RNA-Seq, which is the topic of several recent reviews and research papers [31,76,77,78,79,80,81].

## 4. Modern Approaches to Single-Cell Collection

**Figure 1:**
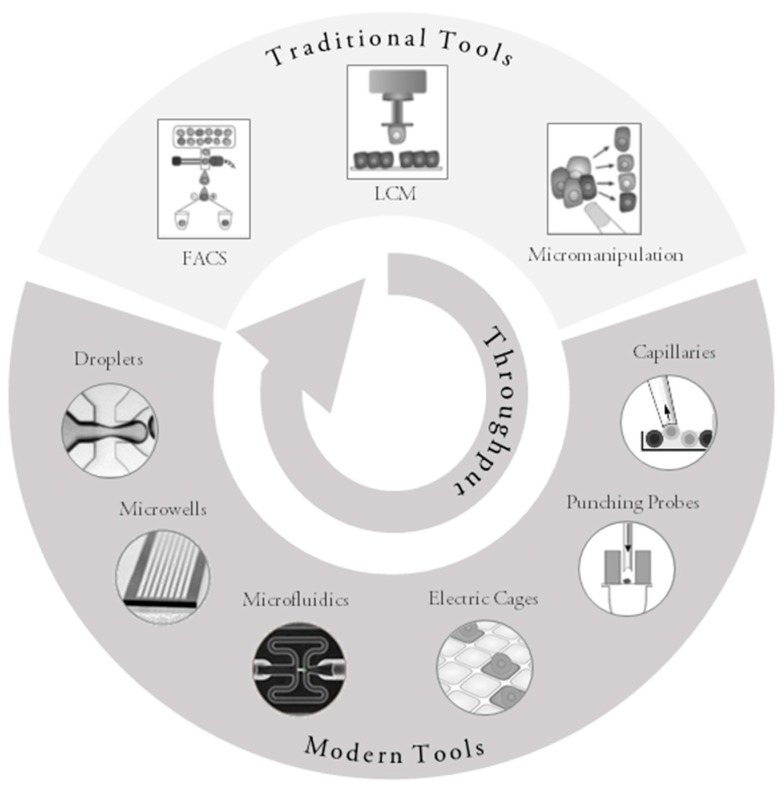
Overview of currently used tools and principles for single-cell collection (adapted and modified from materials provided by the manufacturers and [74,82] with permission from Elsevier). FACS: fluorescence-activated cell sorting; LCM: laser capture microdissection.

Modern approaches to single-cell collection can be classified based on several parameters: the technology/principle for the single-cell collection, throughput, dedicated application if any, or the possibility of visually inspecting and selecting cells (Figure 1). High-throughput approaches usually require a larger starting number of cells; they do not allow for visual inspection, and the possibility to select cells can be limited. Cost per analysis is usually higher. Instruments for routine diagnostics may be CE-IVD marked (European Conformity for In Vitro Diagnostic Medical Devices) and instruments for clinical studies in drug development may be FDA (Food and Drug Administration)-approved, which makes them more expensive. High-throughput instruments process many cells and are suitable for the characterization of rare cells. A large number of cells is prepared in a relatively short time, the cost of analysis per cell is low, and instruments usually provide an end-to-end solution, i.e., a complete analysis including bioinformatics and visualization of the data. Vice versa, low-throughput approaches typically require a longer time to collect cells of interest, there are issues with transfer efficiency (the percentage of cells successfully transferred from the medium into the collection vessel), or the cost per cell is very high, assuming whole-genome amplification (WGA) or whole transcriptome amplification (WTA), which are the usual analyses performed in high-throughput instruments. Instrument manufacturers usually do not provide support for downstream analysis. The main advantage is the ability to select only relevant cells for analysis, minimizing the cost of the experiment. The cells can be selected based on morphology or antibody staining and verified by visual control in a microscope. The low-throughput systems are often customizable and adaptable to any type of downstream analysis [18,19,21,22,23,25,32,33].

There is a continuous effort by producers of new instruments to overcome these barriers. For example, high-throughput instruments offer scalable kits to analyze different starting numbers of cells (ddSEQ Single-Cell Isolator, Bio-Rad (Hercules, CA, USA) and Illumina, (San Diego, CA, USA)), the possibility of performing various types of analysis with a single instrument (genome, exome, transcriptome sequencing, and profiling of immune cells in the Chromium system, 10x Genomics (Pleasanton, CA, USA)), specific or customized panels for targeted RNA/DNA sequencing (Rhapsody Single-Cell Analysis System, Becton, Dickinson and Company (BD, Franklin Lakes, NJ, USA) and Tapestri Platform (MissionBio, San Francisco, CA, USA)), and completely controllable parameters of single-cell collection (Nadia Innovate, Dolomite Bio, Royston, UK). On the other hand, providers of low-throughput solutions try to increase the throughput (new C1 chip for up to 800 cells from Fluidigm (South San Francisco, CA, USA)), offer end-to-end workflows from cell capture to reporting (WTA and WGA kits by Menarini Silicon Biosystems for the DEPArray System (Florence, Italy)), give the possibility of cultivating single cells (CellRaft AIR System, CellMicrosystems (Research Triangle Park, NC, USA)), and even stimulate single cells and analyze their responses - Polaris platform from Fluidigm (South San Francisco, CA, USA). The default protocols may be modified by users, for example, adding enrichment or selection steps (density gradient, FACS, filtration devices, immunomagnetic separation/depletion [19,22,33]); or by applying a low-throughput method combined with a kit including end-to-end analysis (BD precise assays) to process better defined populations of cells. There are even instruments that offer the ability to analyze large numbers of cells, while controlling them visually offers the opportunity to select some for downstream analysis (e.g., ICELL8 Single-Cell System, Takara, Kyoto, Japan).

### 4.1. High-Throughput Devices

An instrument is considered high-throughput if it allows very fast processing of a large number of cells that can be analyzed on the fly. Examples of analyses are RNA-Seq and DNA-Seq. Most instruments employ microfluidics lab-on-chip technologies that generate millions of precisely defined droplets carrying single cells and oligonucleotides serving for the capture of mRNA (e.g., anchored oligo-dT). The droplets may also contain reagents for reverse transcription (RT), to generate cDNA. The droplets can be fused and the library preparation performed in a single tube (usually using gel beads). Alternatively, droplets can serve as capture vessels only. These are subsequently pooled and cDNA is synthetized in bulk (usually using magnetic beads). Libraries for sequencing are then prepared by various workflows including the Drop-seq, inDrop-Seq, SCRB-Seq or companies’ specific protocols [83,84,85]. Based on the principle of mRNA capture, via the poly-A tail, all these protocols generate libraries from relatively short sequences close to the 3′-end of the mRNAs [18,29,32,74,76].

To be able to assign sequencing reads to individual cells in downstream analysis, the capture oligonucleotides contain a sequence that labels all molecules originating from the same cell with a unique barcode. Hence, reads from the same cell share this barcode sequence. Errors may be introduced during the RNA-Seq workflow, most significantly PCR bias, where amplicons are amplified with different efficiency altering the original ratios between cDNA molecules. This can be addressed using extra sequences in capture oligonucleotides that are called unique molecular identifiers (UMI)—a sequence that is unique for each molecule. If two reads with the same target (RNA/cDNA) sequence and UMI are encountered in the computational analysis, they are considered to be PCR duplicates and are merged into a single unique read. This principle allows for digital counting of individual molecules in each cell and is sometimes referred to as digital RNA sequencing [86,87,88].

Although the droplet-based technologies are advanced, they do have limitations. The main drawback is the relatively low RNA capturing efficiency, sensitivity to inhibitors (due to the RT taking place in the very small volume of the droplet), fragility of the droplets, risk of leakage, and low cell capture efficiency when the starting number of cells is limited. Starting with many cells increases the number of doublets (two cells per droplet) and may even clog the microfluidic chip [18,21,73,74,76]. To overcome these issues, some producers exploit other technologies for capturing and processing a large number of cells using arrays with thousands of microwells, each containing a bead coated with oligonucleotides. The downstream analytical steps are like those of the droplet-based platforms.

Here, we provide a brief description of the currently available commercial platforms for high-throughput single-cell collection. A summary is provided in Table 2, where platforms with lower throughput that will be discussed later are also included. Although the low-throughput platforms are dedicated mainly to single-cell collection and do not support any type of downstream analysis, we include them in our review. They represent a supplementary tool for the validation of results measured with the high- and mid-throughput instruments or for rare cell characterization. Since most of the instruments were launched during the last two years, independent comparative data are not available. Our comparisons mainly use data provided by the manufacturers.

#### 4.1.1. Chromium System (10x Genomics)

The Chromium Single Cell 3′ Solution (launched 10/2016) is a scalable platform for the characterization and profiling of hundreds to millions of cells [89]. It utilizes the GemCode technology for barcoding (Gel bead in Emulsion, [99]). The gel beads contain barcoded oligonucleotides that are mixed with RT reagents and cells in oil environment that create the droplets, wherein the cDNA is synthesized. Droplets are then pooled, dissolved, and a cDNA library containing UMI is prepared. The technology can reach 65% cell capture efficiency with a very low doublet rate. Improved second-generation chemistry was recently released, further lowering the doublet rates and increasing the number of transcripts detected. Samples are processed in microfluidics chips, with eight samples per chip in 10–20 min (100–80,000+ cells). The company (Chromium Software Suite, 10x Genomics, Pleasanton, CA, USA) offers an end-to-end solution including the processing, analysis, and visualization of single-cell gene expression data and runs the 10x Community platform, where users can interact and share ideas. The compact instrument can perform genome and exome sequencing, as well as immune repertoire profiling. Based on the number of publications, it is currently the most popular instrument for high-throughput single-cell analysis.

#### 4.1.2. Nadia and RNA-Seq System (Dolomite Bio)

Dolomite Bio was founded in 2016 and introduced recently a new version of its RNA-Seq System called Nadia (launched in November 2017). Like its predecessor, it is a fully automated instrument using the principles described in the DropSeq protocol, one of the seminal works in the single-cell RNA-Seq field [84]. In the instrument, single cells are encapsulated with a single bead having the surface coated with capture oligonucleotides containing UMI sequences. In difference to the previous gel bead technology, the droplets here do not contain reagents for RT. cDNA is synthesized after the collection and breaking of the droplets off the chip. A potential advantage of this approach is the elimination of the risk of RT inhibition in picoliter volumes. On the other hand, in comparison to the gel beads, the cell capture efficiency is lower. The partitioning takes place in a chip for 2–8 samples, which are chilled to improve cell viability (generating up to 48,000 barcoded single cell mRNA libraries). The chip contains cell and beads stirrer, ensuring an even distribution of single cells and beads throughout the run, keeping doublet rates low. The instrument is dedicated to RNA-Seq application, although other applications are possible (e.g., DroNc-Seq or PCR-activated cell sorting (PACS) [90,100,101]). The company does not provide software, since the original Drop-Seq analysis pipeline is freely available (Available online: https://github.com/Hoohm/dropSeqPipe). The instrument may be upgraded to Nadia Innovative, which is a removable module allowing the user to develop new single-cell protocols and applications, use their own reagents, and control all aspects of the droplet formation.

#### 4.1.3. InDrop System (1CellBio)

1CellBio is a recent Harvard University spinout that started an early-access program to its high-throughput instrument, InDrop System, in June 2016. Like with the platforms above, the basic principle of the new instrument is based on the seminal work in the field describing the InDrop-Seq protocol (Indexing in DROPlets, [85]). In this approach cells are first encapsulated with hydrogel microspheres carrying UMI-barcoded primers and then with a lysis buffer and RT mix. After encapsulation, primers are released by ultraviolet radiation (UV) and cDNA is synthesized in each droplet. Droplets are then broken and material from all the cells is amplified for sequencing. The droplets are produced in the chip for six samples, enabling barcoding of 40,000 cells, claiming up to 80% cell capture efficiency. The bioinformatics analysis is based on the freely available inDrop-Seq pipeline (Available online: https://github.com/indrops/indrops). The new version of the system (scheduled for release in mid-2018) should combine flow cytometry and microfluidics technologies.

#### 4.1.4. Single-Cell Sequencing Solution (Illumina, Bio-Rad)

The industry leaders in sequencing and droplet technologies recently (January 2017) introduced their solution for single-cell sequencing. It combines a droplet generator from Bio-Rad, the ddSEQ Single-Cell Isolator (based on the digital PCR instrument QX200), with SureCell WTA 3′ Library Prep Kit from Illumina. Cells are encapsulated into droplets, lysed, captured by barcoded oligonucleotides that include UMIs, and cDNA is synthesized. After pooling and dissolving the droplets, a cDNA library is prepared with the Illumina kit. Droplets are prepared in a disposable cartridge for up to four samples. Scalable kits for different starting numbers of cells are offered (one kit format to process hundreds to low thousands of cells and one kit format for projects that require analysis of up to tens of thousands of cells). End-to-end workflow, including bioinformatics and visualization tools, are provided (SeqGeq™ Analysis Software, Illumina, San Diego, CA, USA).

#### 4.1.5. Tapestri Platform (MissionBio)

Contrary to the aforementioned instruments, the main application of the Tapestri Platform from MissionBio (launched in October 2017) is targeted DNA-Seq. This is ideal when key genes or genomic regions of interest are known. Such an approach may be very useful in precise medicine in immunology and oncology, when information about the co-occurrence of mutations in individual single cells (single nucleotide variant (SNV), indel detection) is needed. The instrument uses a two-step workflow. In the first step, cells are mixed with protease and individual droplets are created. Cell lysis and protease digestion then follow in a standard thermocycler, making the DNA accessible for subsequent amplification. The droplets with cell lysate are then re-loaded into the instrument and mixed with barcoding beads and reagents for amplification of specific regions. The platform analyzes up to 10,000 cells in a single run. The company offers the possibility of customizing panels with targets of interest for single-cell mutation profiling, where up to 100 genomic regions may be analyzed simultaneously. Analysis software is provided. The first application note was made available in November 2017 focusing on acute myeloid leukemia, targeting 19 genes with 40 amplicons.

#### 4.1.6. Rhapsody Single-Cell Analysis System/Resolve (BD)

The new instrument from BD was introduced in September 2017 and is an updated version of the Resolve platform previously made available in a limited number. It is currently the only commercial high-throughput system that is not based on droplet technology. The Rhapsody system is based on arrays of 200,000 microwells with UMI-barcoded magnetic beads capable of capturing up to 20,000 single cells. Captured cells are lysed and mRNA is bound to the beads. The beads are pooled and prepared for library preparation in a single tube. Before lysis, the array is scanned and information about the number of cells, doublets, and empty wells from each sample is obtained. It can help users to decide how many the beads is optimal for library preparation and if the quality of the sample is sufficient for downstream analysis. Remaining beads may be stored for later use. This optimizes the sequencing costs. An additional feature of the Rhapsody system that reduces the cost is the targeted RNA-Seq approach, where only transcripts of interests are amplified in the library preparation. This enhances sensitivity and makes it possible to detect rare molecules that may be missed with whole-transcriptome profiling. The company currently provides several targeted panels, for example, for the profiling of breast cancer, immune cells, and specifically T-cells. Customized panels are also available. Bioinformatics pipeline and visualization tools are provided to enable even inexperienced users to analyze and understand single-cell data. Protein detection on the BD Rhapsody system is planned to become available during the first half of 2018 [102].

### 4.2. Mid-Throughput Devices

The medium-throughput devices fill the gap between instruments designed for the analysis of thousands of cells in a short time and instruments capable of carefully isolating selected tens of cells in hours. They combine their advantages, providing sufficient throughput to allow large-scale studies to survey cellular heterogeneity, but with a focus on a narrow sub-population selected by visual control. This lowers sequencing costs and biostatistics analysis becomes less challenging.

#### 4.2.1. ICELL8 Single-Cell System (Takara)

The ICELL8 Single-Cell System was launched in October 2015 and is a unique instrument combining high throughput with the possibility of visually controlling and actively selecting cells of interests for downstream RNA-Seq analysis [91]. The instrument is a combination of the WaferGen multi-sample nano-dispenser (MSND) with a powerful imaging station. In the first step, the MSND dispenses cells with reagents (typically 50 nL) into a 5184 nano-well chip with pre-printed barcodes. Assuming Poisson distribution, up to 1907 single cells may be captured (notably, the system works with cells 5–100 µm in size). The rapid dispensing (eight samples are dispensed in 15 min), specially formulated diluent, and environmental control of temperature and humidity help sustain cell viability throughout the cell isolation process. After dispensing, the imaging station scans the chip and cells of interest are selected for analysis. Libraries for sequencing may be prepared using the SCRB-Seq protocol combined with Nextera XT DNA Library Preparation Kit (Illumina). Other protocols compatible with the used barcodes may also be used. Bioinformatics analysis software is not a part of the instrument. With this approach, up to 15,000 cells visually selected can be processed in a day.

#### 4.2.2. C1 System and Polaris (Fluidigm)

Fluidigm, with its C1 system for single-cell collection and library preparation, may be considered founders of the modern single-cell field. Since its launch in 2012, the C1 system has been revolutionizing single-cell research. C1 was the first instrument that allowed users to isolate, phenotype, and process single cells for genomic analysis. Cell capture (up to 96 per run), lysis, RT, and cell multiplexing take place in an integrated fluidic circuit (IFC chip) with a complex system of controllable microchannels. The selection of cells in the chip is size-based, currently allowing isolation of cells in the ranges of 5–10, 10–17, and 17–25 µm. In 2015, a new version of the C1 chip was introduced that can process up to 800 medium-size single cells (10–17 µm). Applications of the C1 are broad, as reflected in over 100 published studies, ranging from traditional RNA sequencing and targeted gene expression to whole-genome and exome sequencing, targeted DNA sequencing, epigenetics, and miRNA expression. The versatility of the system increases further with the new C1 Single-Cell Open App IFC chip, which, in combination with the C1 Script Builder, allows users to design customized protocols and methods. Another revolutionary instrument called Polaris was launched in 2015, which is the first and still the only platform that offers a unique unified workflow for active cell selection, cultivation, and molecular analysis (up to 48 cells per run). After capture, individual cells are stimulated by various factors under strictly controlled environmental conditions for up to 24 h and their response is measured by means of next-generation sequencing. Applications are supported by a software solution for the analysis and visualization of the measured data.

### 4.3. Low-Throughput Devices

The last group of instruments for single-cell collection offers the lowest throughput, but with the highest flexibility for downstream applications and the possibility of selecting cells based on different morphological or protein-marker-based criteria. The cells are imaged using powerful microscopes and high-quality images may be collected, making it possible to correlate phenotype with genomic and transcriptomic data. The repertoire of analytical methods is practically unlimited, since the majority of instruments handle only the collection and imaging steps, leaving the choice of downstream analysis to the user. The principles used for cell selection are different, but all are optimized for high transfer efficiency and cell viability. However, in some applications there is a risk of perturbing the expression profiles by the rather long times required to collect the cells of interest.

#### 4.3.1. Puncher Platform (Vycap)

The main advantage of the Vycap Puncher platform is the isolation of rare single cells (e.g., CTC and fetal trophoblasts) in a large starting volume of samples (e.g., 1–10,000 cells in a sample volume of 100 µL–40 mL). To process such large volumes, the sample is filtered through the isolation chip, which is comprised of 6400 microwells (each with a diameter of 70 µm) with a transparent bottom with a single pore (5 µm in diameter). Low pressure is applied across the chip to promote the flow of a cell suspension through the micropores. When a cell enters a well, the pore in its bottom is blocked and the flow is diverted to neighboring wells. In this manner, single cells are sorted in individual wells across the entire chip in a few minutes. After optional cell staining, the chip is transferred to the Puncher system, where multi-colored fluorescence images are acquired and analyzed (based on defined criteria, automatic pre-selection of cells can be made, followed by final manual selection). The selected cells are collected using a specialized punch needle, which is positioned into a well containing a cell and punches out the bottom of the well together with the cell. The tip of the punch needle is shaped such that it only touches the bottom of the microwell but not the cell. The cell, together with the bottom of the punched microwell, is collected in a reaction tube or a plate of choice. The transfer efficiency is very high, ensuring successful collection of more than 95% of the selected cells [92,103,104]. The provider does not offer any reagents for downstream analysis, although compatibility with several commercial WTA and WGA kits has been demonstrated (Repli-G kit of Qiagen and AMPLI-1 kit of Silicon Biosystems, [92,104]).

#### 4.3.2. CellRaft AIR System (CellMicrosystems)

The “punching” technology is also used in the CellRaft AIR System from Cell Microsystems. Although the principle of cell collection is similar, there are some distinct differences. Firstly, the instrument is fully automatized, including a temperature- and humidity-controlled environment. The cells are plated on the array, which contains up to 44,000 microwells (CellRaft Array), in the same manner as a standard tissue culture dish. The cells settle and randomly distribute into microwells. The array is placed in a petri-dish-sized cassette with a reservoir for various media, buffers, and other reagents, allowing culturing of the cells and also testing the influence of different substances (four quad reservoir format). Successful use of the array requires the isolated cells to be adhesive. For work with non-adhesive cells, the array may be coated with an adhesive solution (e.g., CellTak from Corning). After cell capture, the array is imaged and, based on fluorescent signals, cells are selected by two methods: real-time manual selection and by “gating” the population of cells in a cytometric mode. The selected cells are then punched out of the array with a raft (a single microwell with a size of 100 × 100 or 200 × 200 µm). The raft with its attached cell contains paramagnetic nanoparticles and the cells can easily be recovered using a magnetic wand and transported to a 96-well plate or a PCR tube, where they are released by a stronger magnet that is placed under the collection vessel. The gentle mechanism of the release device leaves the cells unperturbed, resulting in high viability and high transfer efficiency [93,94,105].

The principle of cell collection is simple yet very effective and elegant. A manual version of the instrument is also offered (sold as CellRaft System for Inverted Microscopes). The system itself is comprised of a release device with the punching probe and microscope mounted to fit a range of different objective diameters. After manual selection of a cell in the inverted microscope, the raft is released by the punching probe controlled by the release device and manually transferred into a collection vessel using the magnetic wand. The system is attractively priced ($5000, i.e., 10% of the cost of most other instruments). A similar instrument, called QIAscout System, is sold by Qiagen (developed in partnership with CellMicrosystems).

#### 4.3.3. DEPArray NxT and DEPArray System (Menarini Silicon Biosystems)

The DEPArray NxT instrument for single-cell collection (launched in April 2016) combines microfluidic and microelectronic techniques. It uses the ability of a non-uniform electric field to exert forces on neutral, polarizable particles, such as cells, that are suspended in a liquid. This electrokinetic principle, called dielectrophoresis (DEP), can be used to trap cells in DEP “cages” by creating an electric field above a set of electrodes in an array that is in reverse phase with the electric field of adjacent electrodes. When a DEP cage is moved by a change in the electric field pattern, the trapped cell moves with it. These forces are used in the microfluidic cartridge with individually controllable electrodes that create up to 30,000 DEP cages. When a cell suspension is loaded onto the chip, DEP cages are activated and the cells are trapped. The cartridge is scanned in several fluorescent channels to identify target cells, and proper programming of electrodes causes those to move into a “parking” area, where they are deposited. From the parking chamber, individual cells are dispensed into a collection vessel of choice (96 cells per cartridge, or up to 600 cells in pools). Isolated cells are moved gently, without contact or friction, and remain intact and viable for downstream applications, including cell culturing or genome/transcriptional profiling (supported by Ampli1 WGA/WTA kits). An interesting option is the ability to move two different types of cells into the vicinity and study cell–cell interactions [95,96,106].

#### 4.3.4. AVISO CellCelector (ALS)

Capillary-based single-cell collection is employed in the AVISO CellCelector from ALS Automated Lab Solutions (ALS). The instrument combines precise robotic technology with sophisticated image processing software. The harvesting process allows gentle cell uptake directly from the culture plate without pre-treatment. The workflow of the AVISO CellCelector is divided into three steps: image recognition, cell harvesting, and documentation. In the first step, the cells in the culture plate are scanned and automatically analyzed based on pre-defined parameters (intensity of fluorescence, closeness of neighboring cells, etc.). Selected cells are then harvested by mechanical suction within the source plate and subsequently released in a target vessel (culture plate, PCR plate, etc.). The image of the medium before and after collection is acquired to document successful collection process. An advantage of the instrument is the use of common labware, so that no costs for arrays or chips are added. Different sized capillaries are offered, allowing transfer of cell colonies [97,98]. A limitation is lower transfer efficiency when working with cells with strong adhesive properties, and risk of contamination from co-transferred medium (depends of volume), although with proper sample processing the risk is small.

For a summary of the advantages and limitations of the presented single-cell collection platforms, see Table 3.

## 5. Enrichment Technologies

Enrichment technologies are traditionally discussed with a focus on rare cell characterization, i.e., efficiently and specifically capture cells that are present in very low numbers against the background of billions of other cells. Typical example is the isolation of CTCs among billions of red blood cells and millions of leukocytes per milliliter of whole blood [107]. However, enrichment is important in most high-throughput applications, when the goal is to study the heterogeneity of a particular cell type in a tissue composed of many different cell types (e.g., heterogeneity of microglia or astrocytes in brain tissue, [108,109]). Enrichment may help to describe the heterogeneity in greater depth and at much lower cost than when profiling all the cells.

There are several principles for cell enrichment: selection based on physical or morphological features of cells (size, shape, density, electrical polarizability/charge, deformability) and biological properties (presence of surface markers, production of metabolites, and expression of reporter gene). Based on the features, different technologies are used, such as microfiltration, density gradient centrifugation, immunoaffinity (based on antibodies, peptides or aptamers), dielectrophoresis, etc., [110,111,112,113]. Also, traditional FACS can be used. The methods may be applied in their traditional formats, but are often transformed into microfluidic format for the purposes of miniaturization, lower cost, enhanced capturing efficiency, and improved cell viability [112,114]. Most of these methods and technologies were initially developed for capturing of CTCs and are reviewed elsewhere [115,116]. For a broader spectrum of applications, magnetic-activated cell sorting (MACS) based on antibodies bound to superparamagnetic nanoparticles is attractive (Miltenyi Biotec, [117]). Currently, the method is used mainly in the areas of immunology, stem cell research, neuroscience, cancer research, and cardiovascular research [118,119,120].

Despite the many advantages of cell enrichment, there are some drawbacks [23,121]. Inappropriate antibodies, heterogeneous cell properties (depending on cell cycle, cell differentiation, aging), and cell handling (choice of media, temperature, etc.) can introduce bias. Many of these problems are encountered when enriching for CTCs. The reference for CTC enumeration is the FDA-approved CellSearch system, which uses EpCAM antibodies targeting the epithelial cell adhesion molecules. Criticism has aroused in reference to CTCs with lower or no epithelial markers, which are missed by this system, and which may be even more aggressive. Antibody-free strategies have therefore been developed. However, these approaches also struggle with the heterogeneity of CTCs and their physical properties, including the existence of CTC clusters [113,116,122,123]. Similar complexity may be expected for other cell types, particularly when collected under pathophysiological conditions. Any technology for cell enrichment should therefore always be carefully considered and validated to whatever extent is possible.

## 6. Future Perspectives

Single-cell analysis has become a state-of-art method used in different research areas ranging from biology and biochemistry to diagnostics and medicine, but its routine use in research and diagnostic laboratories remains challenging [18,21,73,74]. The main reasons are challenging single-cell collection, a limiting amount of target molecules, the complexity of measurement, the high cost, complex data analysis, and complicated biological interpretation in the context of tissue. For single-cell analysis to become more accessible and reliable, we forecast the following improvements.

Firstly, there is a clear trend to merge enrichment technologies (FACS, etc.) with high-throughput single-cell analysis instruments (already announced by several companies). Being able to actively select only a defined population of cells that are analyzed in high throughput will reduce the need to analyze thousands of cells where only a minority are relevant. This will dramatically reduce the experimental costs and downstream bioinformatic analysis will become less complex. Active selection of cells will also allow for studying heterogeneity among rare cells, which today is possible only with low-throughput methods.

Another challenge in the single-cell field is to control the quality of the collected cells and related pre-analytical variables that may perturb expression profiles (batch effect). In a broader perspective, expression bias induced by sampling procedures and pre-analytical processing is a problem far beyond single-cell profiling; it is the most serious issue for molecular diagnostics [124,125,126]. While it is possible to control the perturbation of expression profiles to some extent (optimizing cell dissociation procedures, selection of fast and gentle method for single-cell collection), tools to assess individual cell quality are missing. It is possible to compare the expression of bulk samples and to create a quality threshold for each cell based on, e.g., the minimal number of genes expressed. However, these approaches are a post hoc type of control, performed when samples have already been processed. The possibility of controlling cell quality before analysis would greatly improve the reliability and cost-effectiveness of single-cell analysis.

Most current methods for single-cell analysis offer the possibility to analyze only one analyte, although measurements of multiple analytes (DNA, mRNA, regulatory RNA, proteins, metabolites) would be much more valuable to describe the complexity of cells [21,25]. A platform that offers such an option will have a competitive advantage (BD recently announced the upgrade of its Rhapsody instrument to allow protein detection [102]). The next level of multianalyte measurements will be in the context of tissue—how cells change expression in response to the presence and proximity to other cells. The first advances in this direction have already been made [127,128].

The data analysis burden increases with throughput. Although many analysis tools are available, their use is limited to a small community of researchers who are successfully combining advanced bioinformatic and statistical skills with knowledge of the studied biological systems. For single-cell analysis to spread to a broader community, user-friendly analysis tools are needed that can be used on personal computers. Analysis of multianalyte measurement will require more sophisticated tools that also aid biological interpretation and prediction of function.

The single-cell field is rapidly developing; revolutionizing approaches are developed almost every day, so many of the challenges of today will be solved tomorrow.

## Figures and Tables

**Table 1:** Comparison of traditional approaches for single-cell collection.

Properties	Micromanipulation	Fluorescence-Activated Cell Sorting	Laser Capture Microdissection
Typical Type of Sample	Viable cells	Viable cells	Non-viable
Throughput	Low	High	Low
Starting Amount of Cells	Low	High	Low
Capability to Capture Rare Cells	Low	High	Low
Analysis	Slow	Fast	Slow
Dissociation	Required	Required	Optional
Visual Inspection (Imaging)	Yes	No (Usually)	Yes
Information about Morphology	Depends on dissociation	No	Yes
Additional Analysis of Sample	No	No	Yes
Contamination Hazard	Yes	No	Yes
Multi-Parameter Analysis	Yes	Yes	No
Laboratory Skills	High	Normal	High
Others	Risk perturbing expression profiles (long collection time, dissociation)	Risk perturbing expression profiles (dissociation, fast flow of medium)	May compromise RNA quality

**Table 2:** Overview of commercially available instruments for single-cell collection and analysis.

Instrument	Chromium System (10x Genomics)	Nadia (Dolomite Bio)	InDrop System (1CellBio)	Illumina Bio-Rad ddSEQ Single-Cell Isolator	Tapestri Platform (MissionBio)	BD Rhapsody Single-Cell Analysis System (BD)	ICELL8 Single-Cell System (Takara)	C1 System and Polaris (Fluidigm)	Puncher Platform (Vycap)	CellRaft AIR System (CellMicrosystems)	DEPArray NxT (Menarini Silicon Biosystems)	AVISO CellCelector (ALS)
Launched in	10/2016	11/2017	6/2016	1/2017	10/2017	09/2017	10/2015	2012 (2015)	8/2015	2017	4/2016	2006
Principles (Reference)	Droplet-based [89]	Droplet-based (Drop-Seq [84])	Droplet-base (InDrop-Seq [85])	Droplet-based	Droplet-based, two-step partitioning [90]	Array of 200,000 microwells, barcoded beads	5184-well chip, pre-printed barcodes, nano-dispensor [91]	Integrated fluidic circuits for up to 800 cells	Array of 6400 microwells with a pore, filtering, punching needle [92]	Array of 44,000 paramagnetic microwells, punching probe, magnetic collection [93,94]	Microfluidic cartridge with 30,000 dielectrophoretic (DEP) cages [95,96]	Capillary-based [97,98]
Main Application	RNA-Seq, DNA-Seq, Immune Repertoire Profiling	RNA-Seq, DroNc-Seq, PACS, open for other	RNA-Seq	RNA-Seq	Targeted DNA-Seq	Targeted RNA-Seq	RNA-Seq	RNA-Seq, DNA-Seq, miRNA-Seq, epigenomics, RT-qPCR	Single-cell collection, rare cell analysis (CTC)	Single-cell collection, tracking cell phenotypes, clonal populations	Single-cell collection, cell–cell interaction	Single-cell collection, transfer of cell colonies
Throughput (# of cells analyzed)	High (>10,000)	High (>10,000)	High (>10,000)	High (>10,000)	High (>10,000)	High (>10,000)	Medium (>1000)	Low-medium (48-800)	Low (<100)	Low (<100)	Low (<100)	Low (<100)
Visual Control	No	No	No	No	No	Yes	Yes	Yes	Yes	Yes	Yes	Yes
Cell Selection	No	No	No	No	No	No	Yes	Yes (C1 size based)	Yes	Yes	Yes	Yes
Starting Amount of Cells	High	High	High	High-medium	High	High-medium	Medium	Medium-low	Low	Medium-low	Medium-low	Medium-low
Flexibility (Own Protocols)	No	Yes (Nadia Innovate)	Unknown	No	Customize panels	Customize panels	Yes	Yes	Yes	Yes	Yes	Yes
Laboratory Skills	Easy	Advanced	Advanced	Easy	Easy	Easy	Easy	Easy	Easy	Easy	Easy	Advanced
End-to-End Solution	Yes	No	No	Yes	Yes	Yes	No	Yes	No	No	No	No
Extra	Intensive support, 10x Community	Sample chilling, cell and beads stirrer, controllable parameters	Early access program—intensive user support	Product from industry leaders, expertize, scalable (kits for different starting number of cells)	Detect mutation co-occurrence, Characterize rare subclones down to 1%	Automated cell counting, archiving, subsampling, promised upgrade to simultaneous protein-detection	Cell selection combined with high throughput	Automatic workflow, staining, library prep, cell stimulation	Established WGA/WTA protocols using Repli-G kit of Qiagen and the AMPLI-1 kit of Silicon Biosystems	CellRaft System for Inverted Microscopes, QIAscout (Qiagen)	Established WGA/WTA protocols using own kits	Customizable, common labware, various harvest modules

**Table 3:** Advantages and limitations of commercially available instruments for single-cell collection and analysis.

Platforms	Advantage	Limitation
Chromium System (10x Genomics)	High cell capture efficiency, easy to operate, end-to-end solution, multiple applications, well established platform, intensive support	High initial cell concentration required, no users modification possible
Nadia (Dolomite Bio)	Open platform, possibility to develop own protocols, multiple applications (PACS, DroNc-Seq)	High initial cell concentration required, lower cell capturing efficiency, no analysis software provided, skills to operate required
InDrop System (1CellBio)	High cell capture efficiency, open platform, possibility to develop own protocols	High initial cell concentration required, no analysis software support, skills to operate required
Illumina Bio-Rad ddSEQ Single-Cell Isolator	Product from industry leaders, easy to operate, end-to-end solution, kits for different starting number of cells	High initial cell concentration required, no users modification possible, single application (RNA-Seq)
Tapestri Platform (MissionBio)	Only platform dedicated to DNA-Seq, easy to operate, customized panels available	Single application possible (DNA-Seq)
BD Rhapsody Single-Cell Analysis System (BD)	Possibility to optimize costs (subsampling, archiving, targeted assays), easy to operate, end-to-end solution, protein detection promised	Single application possible (targeted RNA-Seq)
ICELL8 Single-Cell System (Takara)	Combined high throughput with active cell selection, easy to operate	Bioinformatics analysis not provided, single application (RNA-Seq)
C1 System and Polaris (Fluidigm)	Variable throughput (48–800 cells), multiple applications, customizable protocols, cell stimulation, well established platform, intensive support	Size-based cell selection (C1)
Puncher Platform (Vycap)	Filtering for rare cell capturing, active cell selection, visual control, high transferring efficiency, easy to operate, established WGA/WTA protocols	Low throughput, bioinformatics analysis not provided
CellRaft AIR System (CellMicrosystems)	Multiple applications (cultivation and tracking cell phenotypes, substance testing), active cell selection, visual control, high transfer efficiency, cost-effective manual version available	Low throughput, bioinformatics analysis not provided, adhesive properties of cells expected (although not mandatory)
DEPArray NxT (Menarini Silicon Biosystems)	Active cell selection, visual control, high transfer efficiency, possibility to study cell–cell interaction, established WGA/WTA protocols	Low throughput, bioinformatics analysis not provided; compared to other low-throughput instruments, a high price of consumables (chips)
AVISO CellCelector (ALS)	Active cell selection, visual control, multiple applications (transfer cell colonies), low price for consumables	Low throughput, bioinformatics analysis not provided, skills to operate required, adhesive properties of cells lower transfer efficiency, risk of contamination from co-transferred medium

## Abbreviations

FACS	Fluorescence-activated cell sorting
CTC	Circulating tumor cells
qPCR	Quantitative polymerase chain reaction
RT-qPCR	Reverse transcription quantitative polymerase chain reaction
RNA/DNA-Seq	RNA/DNA sequencing
IHC	Immunohistochemistry
MS	Mass spectroscopy
ddPCR	Digital droplet PCR
LCM	Laser capture microdissection
ICP	Inductively coupled plasma
WGA	Whole genome amplification
CE-IVD	European Conformity for In Vitro Diagnostic Medical Devices
FDA	Food and Drug Administration
WTA BD	Whole transcriptome amplification Becton, Dickinson and Company
RT	Reverse transcription
Drop-seq	Sequencing in droplets
inDrop-seq	Indexing in DROPlets and sequencing
SCRB-seq	Single-Cell RNA Barcoding and Sequencing
UMI	Unique molecular identifier
DroNc-Seq	Droplet single-nucleus RNA sequencing
PACS UV	PCR-activated cell sorting Ultraviolet radiation
SNV	Single nucleotide variant
MSND	Multi-sample nano-dispenser
IFC	Integrated fluidic circuits
DEP ALS	Dielectrophoresis ALS Automated Lab Solutions
MACS	Magnetic-activated cell sorting
EpCAM	Epithelial cell adhesion molecule
